# Anorectal Motility and Sensation Abnormalities and Its Correlation with Anorectal Symptoms in Patients with Systemic Sclerosis: A Preliminary Study

**DOI:** 10.5402/2011/402583

**Published:** 2011-06-06

**Authors:** Hanaa S. Sallam, Terry A. McNearney, Jiande Z. Chen

**Affiliations:** ^1^Department of Neuroscience and Cell Biology, University of Texas Medical Branch, 301 University Boulevard, Galveston, TX 77555-0655, USA; ^2^Department of Internal Medicine, University of Texas Medical Branch, 301 University Boulevard, Galveston, TX 77555-0655, USA; ^3^Department of Microbiology and Immunology, University of Texas Medical Branch, 301 University Boulevard, Galveston, TX 77555-0655, USA

## Abstract

Gastrointestinal (GI) hypomotility and symptoms are common in Scleroderma (SSc) patients yet so far uncorrelated. Eight SSc patients and matched controls were queried about their GI dysmotility symptoms and quality of life (QoL) and underwent anorectal motility and sensory tests. Specific scoring systems were developed for anorectal symptoms and anorectal dysmotility. We found that (1) the SSc patients showed low QoL and marked overall GI symptoms. The most common anorectal symptom was incomplete bowel movement (50%). (2) Compared to normal controls, SSc patients showed impaired anorectal pressures, sensations, and rectal compliance (*P* ≤ .01 for each). (3) The anorectal motility/sensation abnormality score was robustly correlated with the total anorectal symptom score (*r*_*s*_ = .78, *P* = .02). In conclusion, scleroderma patients have impaired anorectal motor and sensory functions, and the abnormality score of these anorectal functions is correlated with the total anorectal symptoms score. These scoring systems may assist clinicians in predicting dysmotility based on patient symptoms.

## 1. Introduction

Systemic sclerosis (SSc, scleroderma) is a multisystemic autoimmune disease characterized by prominent widespread small vessel vasculopathy and endothelial damage with resultant degenerative changes and fibrosis of skin, articular structures, and internal organs [1, 2]. The exact etiology of SSc is unknown, but most studies support the hypothesis that it is an underlying vascular or vasospastic disorder with strong neuropathic and immunologic influences [3].

Gastrointestinal (GI) hypomotility involving the entire gut from the esophagus to the anus is commonly seen in patients with SSc [4]. Nearly 90% of patients with systemic sclerosis have at least one upper or lower GI symptom [5–7]. Anorectal involvement occurs in 50%–70% of SSc patients [8]. Pathophysiological changes of the gut in SSc patients occur in two stages: (a) a neuropathic stage, in which an altered function of enteric nervous system occurs and (b) a myopathic/fibrotic stage, in which smooth muscle is gradually lost or atrophied with progressive fibrosis [8]. Details of the pathophysiological changes in the gut of SSc patients are reviewed elsewhere [9–11]. 

Although gastrointestinal dysmotility and gastrointestinal symptoms are common in SSc, most studies have failed to find any correlations between GI symptoms and upper (esophageal) or lower (intestinal or anorectal) dysmotility [12–15]. Only few studies reported correlations between one upper or lower GI symptom and gastric or anorectal functions [16, 17]. We believe that the difficulty in establishing a correlation between symptoms and GI motility disorders is attributed to the fact that the symptoms and dysmotility are diverse and not particularly focused on a specific organ or symptom. Accordingly, we hypothesized that special scoring systems for overall symptoms and overall motility/sensory abnormalities would be needed in order to correlate symptoms with functional abnormalities. 

The aims of this preliminary study were (1) to characterize abnormalities in anorectal motor and sensory functions in SSc patients, (2) to develop specific scoring systems for anorectal symptoms and anorectal functions, and (3) to study possible correlations between anorectal symptoms and abnormalities in anorectal motor and sensory functions.

## 2. Materials and Methods

### 2.1. Subjects

Two groups comprised of SSc patients (*N* = 8) and normal controls (*N* = 8) were included in the study. The groups were randomly selected and matched for age, gender, race/ethnicity, and weight, as shown in Table 1. The research protocol was approved by the University of Texas Medical Branch (UTMB) Institutional Review Board, and written informed consent was obtained from all subjects before study entry. All SSc patients satisfied the American College of Rheumatology criteria for Scleroderma [18]. To test for skin and other organ involvement by SSc, patients underwent assessment by the Medsger severity index (MSI). Nine subscales derived from the clinical damage of the following: general, peripheral vascular, skin, joint/tendon, muscle, GI tract, lung, heart and kidney, were totaled to constitute MSI scores. MSI scores were from 0–3 where 0 represented no involvement and 3 represented severe or end stage involvement [19, 20]. All patients abstained from prokinetic agents for 72 hours. Controls were selected form healthy, nonsmoking volunteers reporting rare to no GI complaints and were on no medications. Subjects were excluded if they were (1) unable to give informed consent, (2) currently taking prokinetic, anticholinergic or dopaminergic agents which could potentially modify gastric motility, (3) pregnant or preparing to conceive a child, and (4) diabetic.

### 2.2. Experimental Protocol

The subjects underwent a one-day study performed in the Outpatient Clinical GI Motility Laboratory at UTMB. On the study day, following an over night fast, anorectal manometric and sensory tests were performed, and self-report instruments were filled out.

#### 2.2.1. Anorectal Motility/Sensation Test

This test included (1) the measurement of anorectal manometric pressures at resting, and during squeeze and strain, (2) the measurement of rectal anal inhibitory reflex (RAIR) to phasic rectal distension at different pressures, ranging from 10 mL to 60 mL, and (3) rectal sensations to ramp rectal distension, including the threshold distension volumes for first sensation, urge to defecate, and maximum tolerance [13, 15, 21]. The exact procedure is described as follows. 

The evening before the study, subjects were asked to inject one hyperosmolar enema in their rectum (Fleet's enema, C.B. Fleet Company, Inc. Lynchburg, Va, USA), evacuate their bowels and fast overnight. The morning of the study, before coming to UTMB, subjects injected a second enema to ensure complete evacuation of their bowel contents. Upon arrival to UTMB, the subject was positioned lying in the left lateral position with the hips flexed. A lubricated water-perfused manometry catheter with 4 pressure sensors staggered at a longitudinal interval of 1 cm and a rectal distension balloon fixed to its tip was inserted through the anus. The catheter was positioned so that the third sensor (the first sensor is the one that is farthest away from the anus) was in the high pressure zone (the anal sphincter). The catheter was connected to a computerized manometric system (Polygram Net, Medtronic A/S, Kobenhavn S, Denmark). The subject was given 10 minutes to relax and become accustomed to the catheter. The resting sphincter pressure was defined as the highest pressure recorded among the 4 channels. The subject was asked to squeeze the anal sphincter as firmly as possible and maintain it for 25 seconds; this was repeated 5 times. The maximum pressure recorded under these conditions was defined as the maximum squeeze pressure in mmHg. The subject was then asked to strain (or bear down) for 25 seconds; this was repeated 5 times. The maximum intrarectal pressure recorded from the most distal sensor (located farthest away from the anus) during straining was defined as the intrarectal pressure, in mmHg. The residual pressure recorded from the sensors at the high pressure (anal sphincter) zone, during straining, was defined as the residual anal sphincter pressure, in mmHg. 

The RAIR (i.e., relaxation of internal anal sphincter during rectal distension) was elicited by stepwise incremental filling and emptying of the rectal distension balloon (by 10 mL each step), placed in the rectal ampulla. The RAIR threshold, determined as the minimum volume at which the reflex could be elicited was then measured. The threshold of rectal sensitivity defined as the smallest volume of the balloon distension that could be perceived by the subject, was determined by quickly inflating the distending balloon with air (phasic distension) ranging from 10 mL to 60 mL. 

Ramp distension was performed for the assessment of (1) the first sensation to ramp distension, (2) the urge to defecate, which was defined as the least volume to induce an urge to defecate, and (3) the maximum tolerable volume, which was defined as the maximum volume tolerated by the subject by verbal rapport. At the end of the sensation test, the balloon was deflated and the catheter removed. The Polygram Net Software (Medtronic A/S, Kobenhavn S, Denmark) was used to determine various parameters of anorectal motility.

#### 2.2.2. Rectal Compliance and Visceral Sensation Tests

This test was done at the completion of the above anorectal motility/sensation test. A barostat catheter (MUI Scientific, Mississauga, ON, Canada) that was attached with a lubricated polyethylene bag (Pillow Type Rectal Barostat Balloon, capacity 600 mL; MUI Scientific, Mississauga, ON, Canada), was inserted into the rectum [22]. The catheter was positioned so that the middle of the balloon was located approximately 10 cm from the anal verge. The bag was then unfolded by transiently inflating it with 50 mL of air and then deflating it completely. The catheter was connected to a barostat (Synectics Visceral Stimulator, Synectics Medical, Stockholm, Sweden). Following a 10-min adaptation period, tests for visceral sensation and rectal compliance were undertaken. For the determination of visceral sensation (pain or discomfort), automated ramp inflation was used with a step of 20 mL starting from 20 mL, and a step of 50 mL starting from 100 mL to a maximum of 500 mL or the maximal tolerable volume (constant volume mode). The distension at each level was maintained for 45s, followed by a 30s period of no distension. The rectal pressure and intra-balloon volume at each level were recorded. The subject was asked to mark the discomfort level on VAS (0–100 mm) at each level.

The balloon was then immediately deflated and the following protocol was followed to test for rectal compliance. Phasic distension of the balloon was performed in increments of 5 mmHg at a starting baseline of 10 mmHg to a maximum of 60 mmHg, or the maximum tolerance by the subject (constant pressure mode). The distension at each level was maintained for 45s, followed with a 30s period of 5 mmHg distension. The volume at each of the above pressures was recorded. The volume was averaged over the last 30s of each pressure level. A volume-pressure curve was generated for each subject. Rectal compliance, defined as the linear slope of the volume-pressure curve was obtained [22].

### 2.3. Scoring of Anorectal Motility/Sensation Abnormalities

Since motility/sensory dysfunctions in SSc are diverse, a special scoring system was developed to include all abnormalities observed in the anorectal motility and sensation tests, defined as follows: (1) abnormal compliance: <20 mmHg/mL; (2) abnormal RAIR: absence of RAIR at a rectal distension volume of 30 mL or higher; (3) abnormal resting pressure of the anal sphincter: <40 mmHg, (4) abnormal squeeze pressure of the anal sphincter: <80 mmHg; (5) abnormal sensations, including (a) first sensation threshold: >30 mL, (b) urge to defecate threshold: <80 mL and (c) maximum tolerable volume threshold: <200 mL [23, 24]. Each of these abnormalities was assigned one point and the total score was derived for each subject, with a possible maximum score of 7.

#### 2.3.1. Questionnaires of Quality of Life and GI Symptoms

**Health-Related SF-36 Instrument (Version 1.0)**

This measures nonorgan specific quality of life (QoL) and has been validated in SSc patients, normal populations and in other health conditions [25–29]. Raw SF-36 domain scores and physical component summary (PCS) and mental component summary (MCS) from our study were converted to version 2.0, using a 2.0 conversion kit (SF Health Outcomes Scoring Software, QualityMetric Incorporated, Lincoln, RI, USA). The converted data can then be compared with normative data for the US population and other study populations. The recall on the SF-36 items was 4 weeks.

**GI Dysmotility Questionnaire (GIDQ)**

This was designed to assess GI related symptoms and GI-related quality of life, based on previous literature [30, 31]. The GIDQ or parts of it had been used in previous studies [16, 17, 32, 33] and is presented elsewhere [33]. We designed and used the GIDQ as at the time of this study, a validated GI dysmotility symptoms questionnaire for SSc patients was not available. The GIDQ includes 48 items including questions on GI specific symptoms, including presence, frequency and intensity of symptoms and their effects on the subject's normal daily activities during the last week. This questionnaire also queried issues related to perceived functioning and quality of life. Patient responses included “yes or no” answers and scoring on visual analog scales (VAS, range = 1–7) for frequency of symptoms (days/week) and subjective intensity of the symptom (0–100 mm).

### 2.4. Scoring of Anorectal Symptoms

To investigate possible correlation between anorectal motility/sensory abnormalities and anorectal symptoms, two overall scoring systems were used to assess anorectal symptoms, inferred from the symptoms queried in the GIDQ, namely, the following: fecal incontinence, hard stool, rectal fullness, incomplete bowel movement and bleeding due to straining. The first scoring system was based on the frequency of symptoms (the anorectal symptom frequency score), in which the weekly frequency of each of the 5 anorectal symptoms was summated for each subject. The maximum score was 35/subject (5 symptoms, 7 days a week each). The second scoring system was based on the prevalence of symptoms (the anorectal symptom prevalence score), in which each symptom was graded as 0 for absence and 1 for presence. The maximum score was 5/subject.

### 2.5. Statistical Analysis

All data are presented as mean ± SEM. Unpaired Student's *t*-test was used to assess the difference in each of parameters of anorectal motility between the SSc patients and the normal controls. The strength of the association between SSc and the absence of cutaneo-anal reflex was expressed as odds ratio (OR), with 95% confidence interval, calculated by Chi Square analysis (GraphPad Prism-La Jolla, CA, USA). Correlation was assessed using Spearman Rank Order analysis (SPSS 17.0 for Windows—Chicago, Ill, USA). A *P* value ≤.05 was considered significant.

## 3. Results

### 3.1. QoL and GI Symptoms

The demographics of the study populations are shown in Table 1. There was a good match in age, sex, and race between the patients and the healthy controls. At the time of the study, no patient was using laxatives, and only one was under corticosteroid treatment. SSc patients had low QOL and marked overall GI symptoms. Self-reported prevalence of anorectal symptoms for the SSc patients are shown in Table 2. All SSc patients reported at least one anorectal symptom and 50% of the SSc patients had two or more anorectal symptoms at least once a week. Self-reported frequency of anorectal symptoms for the SSc patients are shown in Table 3.

### 3.2. Anorectal Motility

Table 4 shows the mean values of measurements reflecting anorectal functions in the SSc patients and normal controls. The cutaneo-anal reflex was absent in 38% of patients (OR = 3.7; *P* = .03). The resting and squeeze pressures of the anal sphincter, and the intrarectal pressure during straining were all lower in the SSc patients than the normal controls (*P* ≤ .01 for each comparison). The threshold volume of rectal distension to induce RAIR was not significantly different in the SSc patients compared to the normal controls (*P* = .1); however, in one SSc patient, the RAIR was not elicited even at 60 mL. This is the same patient that showed highest scores in symptom frequency, symptom prevalence and anorectal motility/sensation abnormality scores. All anorectal sensations had shown to be decreased in the SSc patients compared to the normal controls. There was significant decrease in the threshold for the first sensation by 39%, the urge to defecate by 21% and the maximum tolerable volume by 31% in the SSc patients compared to the normal controls. 

**Figure 1:**
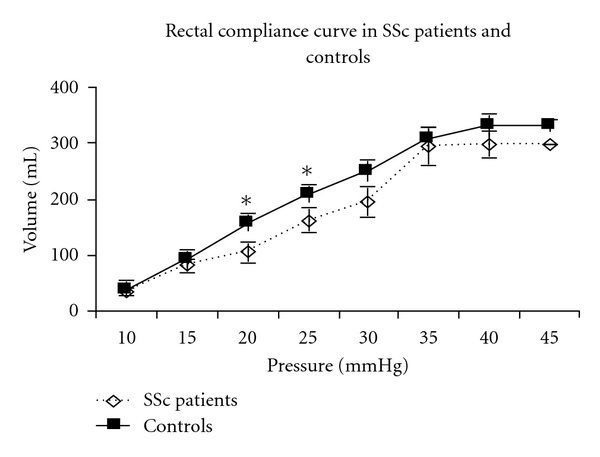
Rectal compliance curve in SSc patients versus controls. As shown, rectal compliance was significantly lower versus controls. In SSc patients, rectal volumes were significantly lower versus controls at 20 mmHg, and 25 mmHg (*P = .04 and.05, resp.).

Rectal compliance was significantly lower in the SSc patients compared to the normal controls. This is shown by the reduced *β* (representing the half-max volume on the pressure-volume curve, a 24% reduction, *P* = .05), *P*_1/2_ (representing the overall shape of the compliance curve, *P* = .03) and *P*_max_ (representing the maximum tolerated pressure, a 24% reduction, *P* = .05).  Figure 1 shows rectal compliance curves in the SSc patients and the normal controls. Rectal volumes were significantly lower to the applied balloon pressures in the SSc patients compared to the controls at 20 mmHg (a 33% reduction, *P* = .04) and 25 mmHg (a 23% reduction, *P* = .05). There were no significant differences between the SSc patients and the normal controls in the reported VAS scores (for abdominal pain and urge to defecate) at any balloon distension level up to 30 mmHg. It is worth noting that 5 SSc patients could not tolerate rectal balloon distension at any pressures >30 mmHg, thus the reporting for the urge to defecate and abdominal pain due to rectal distension was only obtained at a pressure of 30 mmHg or lower in these patients. Three patients had modestly higher VAS scores for pain and urge to defecate than the normal controls at pressures >than 35 mmHg (*P* = NS, not shown).

### 3.3. Correlation of Anorectal Motor and Sensory Abnormalities and Anorectal Symptoms

**Figure 2:**
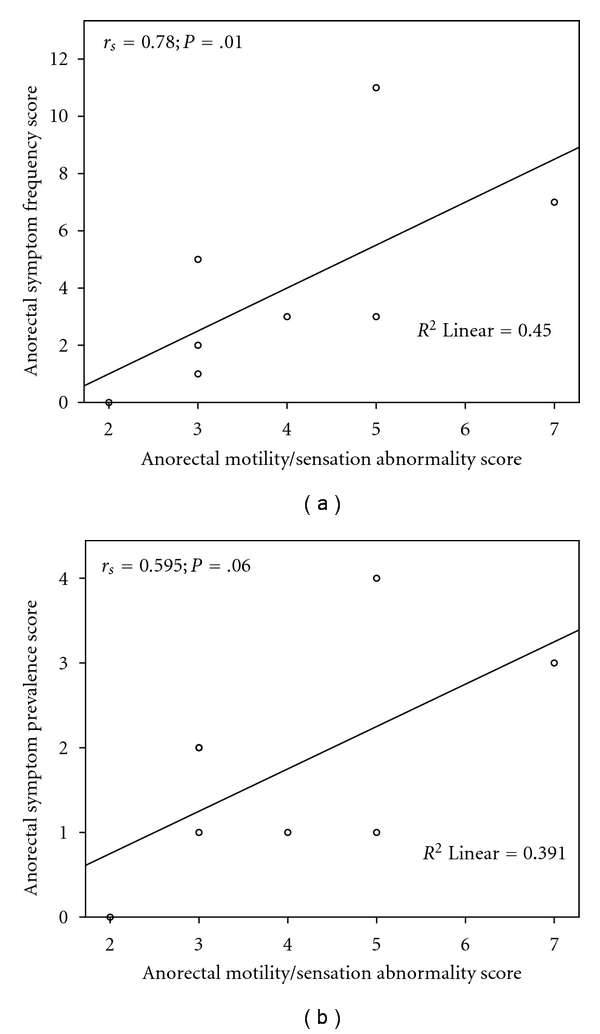
The correlation between anorectal motility/sensation abnormality score and the anorectal symptom scores in the SSc patients. (a) Correlation of the anorectal motility/sensation abnormality score with the anorectal symptom frequency score in the SSc patients (*r* = 0.78; *P* = .01). (b) Correlation of the anorectal motility/sensation abnormality score with the total anorectal symptom prevalence score in the SSc patients (*r* = 0.595; *P* = .06).

Based on our scoring system, all of the SSc patients (100%) had anorectal motility/sensation abnormalities (Table 5).  Figure 2 demonstrates the correlation between anorectal motility/sensation abnormality score and the anorectal symptom scores in the SSc patients. The anorectal motility/sensation abnormality score were significantly and robustly correlated with the anorectal symptom frequency score (*r*_*s*_ = 0.78; *P* = .01—Figure 2(a)) and marginally correlated to the anorectal symptom prevalence score (*r* = 0.595; *P* = .06—Figure 2(b)). Interestingly, in SSc patients, the abdominal pain VAS score was inversely correlated with intrarectal balloon volume at 20 mmHg (*r*_*s*_ = −0.53; *P* = .04) and 25 mmHg (*r*_*s*_ = −0.49; *P* = .05), suggesting that reduced rectal compliance might cause abdominal pain. 

There was a marginal correlation between the anorectal motility/sensation abnormality score and the overall GIDQ scores (*r*_*s*_ = 0.69; *P* = .058). There was no correlation between the anorectal motility/sensation abnormality score and QoL (*r* = −0.3; *P* = .5 and *r* = −0.1; *P* = .8 for PCS and MCS, respectively).

## 4. Discussion

This study demonstrated the following: (1) patients with SSc showed severe anorectal motility and sensation abnormalities, including reduced resting and squeeze pressures of the anal sphincter, reduced rectal pressure during strain, impaired rectal compliance and reduced first sensation threshold, urge to defecate, and tolerance to maximum rectal distension and (2) the anorectal motility/sensation abnormality score was robustly correlated with the anorectal symptom frequency score and was marginally correlated with the anorectal symptom prevalence score in SSc patients.

In the present study, we were able to prove a correlation between the anorectal motility/sensation abnormality score and the anorectal symptom frequency score. To the best of our knowledge, our study was the first to demonstrate a significant correlation between the anorectal motility/sensation abnormality score and the anorectal symptom frequency score. This result was based on calculations deduced form our scoring systems, and it suggests that the overall anorectal symptoms may be predictive of overall anorectal motility/sensation dysfunction in SSc patients. Researchers have been eagerly searching for a correlation between anorectal symptoms and anorectal dysmotility to be able to predict underlying anorectal dysmotility from SSc patient symptoms. Though the majority were generally unsuccessful, however, in SSc patients, a negative correlation between maximum tolerable volume and diarrhea [34] and a positive correlation between impaired RAIR and fecal incontinence [35] were reported. Recently, we have reported a positive correlation between the frequency of heartburn, an upper GI symptom, and the changes in gastric myoelectrical activity, a gastric function, as assessed by electrogastrography [16]. 

Anorectal symptoms were quite common among SSc patients. Most SSc patients self-reported more than one anorectal symptom or experienced symptoms >2 times/week. Incomplete bowel movement was the most prevalent symptom in the SSc patients. 25% of SSc patients reported to have fecal incontinence; this was comparable to other reports in which fecal incontinence ranges between 20%-21% [34, 36]. Other researchers reported a higher percentage of incontinence (87.5%); however, this was in an SSc population in which 50% suffered from associated rectal prolapse [24]. Despite high prevalence of anorectal symptoms in SSc patients, patients are often reticent or too embarrassed to report these symptoms to their treating physicians, so these symptoms are often undertreated [37]. Physicians must be aware of the prevalence and frequency of anorectal symptoms in order to elicit ongoing GI symptoms, so they can target the patient problem with appropriate therapies. Queries regarding changes in daily activities due to GI-related problems may unmask significant daily compromises to compensate for GI dysfunctions. Our scoring system offers a simple, feasible way to score patient symptoms to predict underlying anorectal dysmotility.

It is known that SSc patients with severe GI involvement experience considerably reduced QoL in physical and social dimensions [17, 25], with increased mortality of up to 85% within 9 years, compared to patients with minor GI complaints [38]. The Medical Outcome Survey Short-Form 36 (SF-36) questionnaire has been used to assess quality of life issues and the systemic involvement [25]. This questionnaire can serve as an excellent tool for evaluation of quality of life in this particular group of patients, hence our use in the current study. 

On the contrary, to date, there are no validated GI symptom questionnaires targeting SSc patients which can elucidate correlative links between GI-related symptoms and GI dysmotility by conventional tests. Generally, GI symptoms have not been predictably correlative to the histologic or physiologic severity of GI dysmotility or morbidity in SSc [8, 39, 40]. We have written the GIDQ questionnaire for GI symptoms that includes frequency of symptoms, intensity of symptoms and quality of life issues. Our recent studies showed that it can be used to determine the effect of treatment on GI symptoms as well as to correlate symptoms to gastric dysmotility [16, 17]. The GIDQ has been published elsewhere [33]. 

The uniqueness of this study lies in our detailed comprehensive study of the anorectal functions. While other researchers focused on one aspect of anorectal functions, we provided a complete study of motor, sensory and tonic functions of the anorectum in SSc patients. We have reported decreased anorectal functioning by rectal pressures, sensation and compliance, supporting neurologic and myogenic contributions to GI dysfunction in the SSc patients. The cutaneo-anal reflex was significantly decreased in SSc patients and three patients had a negative reflex, implicating a possible autonomic neuropathy, as deduced form the significant association of the odds ratio. It is evident that autonomic dysfunction has been well recognized in SSc patients [17, 41, 42]. Anorectal manometric measurements were decreased in the SSc patients compared to normal controls. Low resting pressure has been reported in 62.5% of SSc patients; these results were comparable to other reports [12]. In the SSc patients, though the maximum squeeze pressure, a function of the skeletal external anal sphincter, was lower than normal control, it remained within normal range. This was in agreement with previous reports [12, 35, 36, 43–45] and is thought to be due to the selective fibrotic effect of scleroderma on the smooth internal anal sphincter [35]. RAIR, an intrinsic reflex to relax the internal anal sphincter in response to rectal distension was detected in 50% of our SSc patients, comparable to other reports [5, 24]. Only one patient showed no RAIR, even at the highest distension volume. Absence, decrease or paradoxical RAIR is common in SSc patients [36, 43, 44, 46]. Higher percentage of impaired or absent RAIR has been also reported [35, 43–45]. Impaired or absence of RAIR is an indicator of intrinsic rectal neural reflex disruption, similar to the early reported duodenal neural reflex disruption in SSc patients [47]. The thresholds for all sensations were lower in the SSc patients than the normal control. In particular, maximum tolerable volume, an index of the extent of involvement of GI manifestations in SSc [34], was significantly lower in SSc patients in accordance of previous reports [24, 36, 43, 48–50]. This reduction in the maximum tolerable volume reflected lower compliance of the anorectum and was verified by actual barostat measurements for rectal capacity and compliance in the current study. 

In the progression of pathological changes in the GI tract in SSc, it is hypothesized that the neurogenic stage occurs first, followed by the myopathic/fibrotic stage. We have found three types of anorectal abnormalities in the SSc patients: (1) reduced contractility or tone/pressure, (2) reduced sensation, and (3) reduced compliance. Although we cannot determine which of the pathological processes is responsible for the anorectal abnormalities noted in the current study, we suggest that the reported reduction in anorectal contractility is possibly attributed to myopathy, while the reduced sensation may be attributed to collagen deposition and neuropathy or to muscle atrophy and fibrosis, as suggested by Whitehead et al. [34]. The reduced compliance is probably attributed to both the loss of rectal elasticity, the resultant of collagen infiltration in the rectal lamina propria, as shown in the autopsies of SSc patients [48] or by loss of myotonic tone or by neuropathy. However, reduced compliance with intact sensation (as inferred from the presence of abdominal pain in response to balloon distension) may suggest that the neurogenic stage may be subsequent to the fibrogenic stage or possibly that if neurogenic processes are the initiating event, then the motor neuropathy occurs before sensory deficits. It is unknown if the pathological stages of SSc GI dysfunction occur in tandem or subsequently with increased GI related symptoms or decreasing quality of life. The correlation of the presence of abdominal pain with balloon distensions at 2 discrete pressure points suggests they may occur in tandem. 

In summary, we have developed an anorectal motility/sensation abnormality scoring system for SSc patients, and this special score is robustly correlated with the anorectal symptom frequency score and marginally correlated with the anorectal symptom prevalence score. The scoring system proposed in this study may be useful in predicting underlying anorectal motility/sensation abnormalities in SSc patients.

## Figures and Tables

**Table 1:** Demographic characteristics of the study populations. Comparisons between SSc patients and matched normal controls are performed by Student's *t*-test. Data are shown as mean ± SE, (range of values), or percentages. Mean GI symptoms: mean of total GI symptoms scored as prevalence, self-reported on the GI symptoms questionnaire by SSc patients or normal controls. Daily GI meds: the percentage of subjects per group that are on daily medication (s) to treat GI symptoms. SF-36 PCS and SF-36 MCS: physical component summary score and mental component summary score derived from the SF-36 questionnaire.

Variables	SSc patients (*N* = 8)	Controls (*N* = 8)	*P* value
Age, years, range	59 ± 3 43–68	53 ± 3 45–61	.07
Females, % (number)	75% (6)	88% (7)	.3
Postmenopausal, % (number)	83% (5)	86% (6)	.5
Parity in females (average)	2.3	2.6	.3
Pelvic surgeries (average)	0.5	0.1	.1
Caucasian % (Number)	88% (7)	75% (6)	.3
Mean weight (lbs)	153 ± 11	160 ± 9	.3
Mean disease duration (yrs)	7.6 ± 2	N/A	
Medsger Severity index score	5.6 ± 1	N/A	
Mean GI symptoms	17 ± 3	0.5 ± 0.3	<.01
Daily GI meds %	88%	0%	<.01
SF-36 PCS	38 ± 3	54 ± 3	.001
SF-36 MCS	48 ± 3	57 ± 2	.02

**Table 2:** Prevalence of anorectal symptoms self-reported by the SSc patients. +: Yes; −: No; BM: bowel movement.

	Hard stools	Incomplete BM	PR bleeding	Rectal fullness after BM	Stool incontinence	Total anorectal symptom score
Patient 1	−	−	+	−	−	1
Patient 2	−	−	−	−	−	0
Patient 3	−	−	−	−	+	1
Patient 4	−	+	−	+	−	2
Patient 5	−	+	+	−	−	2
Patient 6	+	+	−	+	−	3
Patient 7	+	+	−	+	+	4
Patient 8	+	−	−	−	−	1
Sum	3	4	2	3	2	
Percentage	37.5%	50%	25%	37.5%	25%	

**Table 3:** Frequency (days/week) of anorectal symptoms self-reported by the SSc patients. BM: bowel movement.

	Hard stools	Incomplete BM	PR bleeding	Rectal fullness after BM	Stool incontinence	Total anorectal symptom score
Patient 1	0	0	3	0	0	3
Patient 2	0	0	0	0	0	0
Patient 3	0	0	0	0	3	3
Patient 4	0	1	0	1	0	2
Patient 5	0	1	4	0	0	5
Patient 6	5	1	0	1	0	7
Patient 7	7	1	0	1	2	11
Patient 8	1	0	0	0	0	1

**Table 4:** Anorectal measurements for the SSc patients and the controls.

Variables	SSc patients	Controls	*P* value
*Manometry*			
Max. anal resting pressure (mmHg)	27.5 ± 5	48.8 ± 4	.002
Max. squeeze pressure (mmHg)	71.8 ± 14	97.5 ± 5	.05
% increase in anal sphincter pressure during squeeze (mmHg)	32.3 ± 5	50.6 ± 5	.01
Maximum intrarectal pressure (mmHg)	45.5 ± 6	67.5 ± 5	.01
Residual anal sphincter pressure during straining (mmHg)	19.6 ± 8	29.5 ± 6	.1
Least volume to induce RAIR (mL)	25.0 ± 6	17.5 ± 3	.13
Threshold for 1st sensation (mL)	23.8 ± 5	38.8 ± 6	.03
Threshold for Urge to defecate (mL)	73.8 ± 13	93.8 ± 7	.04
Maximum tolerable volume (mL)	108.8 ± 14	156.3 ± 13	.01
*Compliance *			
Kappa (instantaneous slope of the curve)	1.1 ± 0	1.2 ± 0	.4
Beta (overall shape of the curve)	26.4 ± 4	34.8 ± 3	.05
*P*_1/2_ (half-max volume on pressure-volume curve - mmHg)	16.8 ± 2	22.0 ± 2	.03
*P*_max_ (maximum tolerated pressure - mmHg)	33.1 ± 2	42.1 ± 4	.05

**Table 5:** Anorectal motility/sensation abnormality scoring system. +: Yes; −: No.

			Abnormal Pressures		Abnormal sensations			
	Abnormal compliance	Absence of RAIR up to 30 mL	Resting	Squeeze	First sensation threshold	Urge to defecate	Maximum tolerable volume	Anorectal motility/sensation abnormality scores
Patient 1	+	−	+	−	−	+	+	4
Patient 2	−	−	+	+	−	−	−	2
Patient 3	+	+	+	−	−	+	+	5
Patient 4	+	+	−	−	+	−	−	3
Patient 5	−	−	−	+	+	+	−	3
Patient 6	+	+	+	+	+	+	+	7
Patient 7	+	−	+	+	−	+	+	5
Patient 8	+	+	−	−	+	−	−	3
Sum	6	4	5	4	4	5	4	
Percentage	75%	50%	62.5%	50%	50%	62.5%	50%	
